# Diagnosis and management of postpartum hemorrhage and intrapartum asphyxia in a quality improvement initiative using nurse-mentoring and simulation in Bihar, India

**DOI:** 10.1371/journal.pone.0216654

**Published:** 2019-07-05

**Authors:** Rakesh Ghosh, Hilary Spindler, Melissa C. Morgan, Susanna R. Cohen, Nilophor Begum, Aboli Gore, Tanmay Mahapatra, Dilys M. Walker

**Affiliations:** 1 Institute for Global Health Sciences, University of California, San Francisco, San Francisco, CA, United States of America; 2 Department of Pediatrics, University of California, San Francisco, San Francisco, CA, United States of America; 3 Maternal, Adolescent, Reproductive, and Child Health Centre, London School of Hygiene and Tropical Medicine, London, United Kingdom; 4 College of Nursing, University of Utah, Salt Lake City, UT, United States of America; 5 CARE India, Patna, Bihar, India; 6 Department of Obstetrics and Gynecology and Reproductive Services, University of California, San Francisco, San Francisco, CA, United States of America; Cincinnati Children's Hospital Medical Center, UNITED STATES

## Abstract

**Background:**

In the state of Bihar, India a multi-faceted quality improvement nurse-mentoring program was implemented to improve provider skills in normal and complicated deliveries. The objective of this analysis was to examine changes in diagnosis and management of postpartum hemorrhage (PPH) of the mother and intrapartum asphyxia of the infant in primary care facilities in Bihar, during the program.

**Methods:**

During the program, mentor pairs visited each facility for one week, covering four facilities over a four-week period and returned for subsequent week-long visits once every month for seven to nine consecutive months. Between- and within-facility comparisons were made using a quasi-experimental and a longitudinal design over time, respectively, to measure change due to the intervention. The proportions of PPH and intrapartum asphyxia among all births as well as the proportions of PPH and intrapartum asphyxia cases that were effectively managed were examined. Zero-inflated negative binomial models and marginal structural methodology were used to assess change in diagnosis and management of complications after accounting for clustering of deliveries within facilities as well as time varying confounding.

**Results:**

This analysis included 55,938 deliveries from 320 facilities. About 2% of all deliveries, were complicated with PPH and 3% with intrapartum asphyxia. Between-facility comparisons across phases demonstrated diagnosis was always higher in the final week of intervention (PPH: 2.5–5.4%, intrapartum asphyxia: 4.2–5.6%) relative to the first week (PPH: 1.2–2.1%, intrapartum asphyxia: 0.7–3.3%). Within-facility comparisons showed PPH diagnosis increased from week 1 through 5 (from 1.6% to 4.4%), after which it decreased through week 7 (3.1%). A similar trend was observed for intrapartum asphyxia. For both outcomes, the proportion of diagnosed cases where selected evidence-based practices were used for management either remained stable or increased over time.

**Conclusions:**

The nurse-mentoring program appears to have built providers’ capacity to identify PPH and intrapartum asphyxia cases but diagnosis levels are still not on par with levels observed in Southeast Asia and globally.

## Introduction

Globally, an estimated 275,000 maternal deaths and 2.7 million neonatal deaths occur annually, a quarter of which occurs in India [1, 2]. Hemorrhage, the leading cause of maternal mortality accounted for 27% of all deaths globally and 38% in India [3, 4]. Intrapartum asphyxia is the second important cause, accounting for 11% and 19%, of all neonatal deaths globally and in India, respectively [2, 5]. Further, a third of all neonatal deaths globally [6] and in India [7] occur within 24-hours of birth. Thus, interventions aimed at improving intrapartum and immediate postnatal care could significantly impact neonatal and maternal survival.

A critical step towards preventing maternal and neonatal mortality is timely diagnosis and management of postpartum hemorrhage (PPH) and intrapartum asphyxia, which remains largely underdiagnosed in primary care facilities in India [8, 9]. Skilled health personnel, who attend 71% of all deliveries worldwide and 79% in India, need to be able to identify and manage such complications [10, 11]. In fact, estimates suggest that basic neonatal resuscitation (NR) including drying and stimulating, repositioning, clearing airways and positive pressure ventilation (PPV), could prevent about 30% of intrapartum-related neonatal deaths [12].

The Government of India initiated a program in 2005 to increase institutional deliveries with the expectation that skilled attendants are better able to identify and manage maternal and neonatal complications, thereby saving lives [13]. However, in the state of Bihar, where the population is predominantly rural [14], despite an increase in institutional delivery, concomitant reduction in neonatal mortality was not observed [2], suggesting sub-optimal quality of care in these facilities. Indeed, studies from Bihar report that providers lack essential clinical skills, and facilities lack trained staff and adequate infrastructure [15, 16].

A nurse-mentoring program including integrated simulation training targeting individual and team performance was implemented in Bihar with the overall aim of improving the quality of facility-based care [17]. Previous reports have demonstrated effectiveness of this intervention, implemented on a smaller scale, to increase use of evidence-based practices (EBP) for both intrapartum and neonatal care among normal deliveries [18, 19]. We hypothesized that the nurse-mentoring program also built the providers’ capacity to identify and manage maternal and neonatal complications. The objective of this analysis was to examine changes in diagnosis and management of PPH and intrapartum asphyxia during a mobile nurse-mentoring program in 320 Basic Emergency Obstetric and Neonatal Care (BEmONC) facilities in Bihar, India. The SQUIRE 2.0 guidelines to report quality improvement studies were used [20].

## Methods

### Setting

In 2011, CARE India, a non-governmental organization, collaborated with the Government of Bihar to implement a pilot program in eight districts [18]. Promising results from the pilot phase [18, 19] led to scale-up to all 38 districts in Bihar, covering an estimated 110 million population, as *Apatkaleen Matritva evam Navjat Tatparta* (AMANAT), meaning ‘emergency obstetrical and neonatal readiness’ in Hindi. AMANAT was a multi-faceted quality improvement nurse-mentoring program to reduce maternal and neonatal mortality by improving provider skills in normal and complicated deliveries. Other key components included support for positive changes in infrastructure and management, infection control, hazardous waste disposal, and creating and maintaining a newborn care corner in public health facilities.

The AMANAT program was implemented in four phases between May 2015 and January 2017 at 320 high volume, BEmONC facilities at the primary care level (80 facilities per phase (P), P1 –May to October 2015, P2 –September 2015 to May 2016, P3 –Nov 2015 to June 2016 and P4 –June 2016 to Jan 2017). Due to administrative limitations, only facilities with adequate readiness in terms of infrastructure and management were included. In Bihar, BEmONC facilities serve twice as many people than federally mandated, often with limited resources to effectively diagnose and manage obstetric and neonatal emergencies [14]. Only vaginal deliveries are conducted in these facilities, attended by auxiliary or general nurse midwives (ANMs and GNMs).

### Intervention

In each phase, a pair of nurses (mentors) were assigned four facilities to conduct on-site mentoring of labor room nurse mentees [21]. Mentor pairs visited each facility for one week, covering four assigned facilities over a four-week period. The mentor pairs returned for subsequent week-long visits once every month for seven to nine consecutive months. In other words, facilities received one week of mentoring in a month for 7–9 consecutive months. The mentors engaged in a variety of activities including skill demonstrations, didactic sessions, high-fidelity simulation and bedside mentoring during actual patient care.

An integral part of the nurse-mentoring program was PRONTO International’s (http://prontointernational.org) simulation and team training. The simulation and team training curriculum was tailored to address local contextual needs and incorporated in the AMANAT program since the outset. The three components of the PRONTO curriculum were: (1) realistic human-centered in-situ simulation scenarios of normal and complicated deliveries, including scenarios with simultaneously occurring emergencies, to promote use of EBPs, (2) efficient teamwork and communication (T&C) among providers and (3) increasing provider awareness around person-centered maternity care [21, 22]. Simulations were conducted in providers’ usual work settings utilizing a maternal actor wearing PartoPants (a hybrid low-tech birth simulator) [22], and a NeoNatalie infant mannequin, nurses from the facilities acted as the patient to gain in-sight into the patient experience. A unique aspect of PRONTO’s simulation is use of a maternal actor instead of a mannequin. PRONTO trained all nurses to facilitate and video-record simulations, conduct video-aided debriefings after simulations and perform rapid debriefings after live deliveries.

The T&C component focused on building collaborative environment among mentees. It included structured team-building activities as well as integration of specific communication techniques, including ‘think out loud,’ ‘call back,’ ‘call out,’ ‘SBAR’ (Situation, Background, Assessment, Recommendation), and debriefing (adapted from the TeamSTEPPS curriculum) [23]. These activities provided mentees with an opportunity to practice technical and non-technical competencies required to manage a variety of obstetric and neonatal complications as a team, even as a very small team.

The Institutional Review Board of the Indian Institute of Health Management Research in Jaipur, India (date–June 27, 2015) and the Committee for Human Research at the University of California San Francisco approved the study (date–May 20, 2015). Study ID# 14–15446.

### Data collection systems

We used two data sources, which were collected and maintained by CARE India- the Facility Information System (FIS) and direct observation of deliveries (DOD). The FIS system was used to record data for the weeks of mentoring and DOD was conducted before and after mentoring. FIS was a web-based system, which provided information on deliveries and mentoring activities. Data were collected daily by the mentors during each of their week-long visits and entered directly into the system. The mentors obtained the daily data on all deliveries that occurred during the day using observation and facility registers and cross-checked with the staff when necessary. FIS data were collected only for the weeks when mentors were present in the facilities for mentoring and not for the other weeks. Delivery data included patient demographics, delivery mode, obstetric and neonatal complications, intrapartum management and discharge dispositions. Mentoring data included date, time and topics covered in each session, number and characteristics of simulations performed and staff attendance. The second source of data was DOD, collected by clinically trained nurses who observed deliveries between 9 am and 5 pm over the week immediately before and after the intervention. At baseline, when mentoring had not yet started, nurses observed deliveries in the facilities they later mentored, but for endline, they observed deliveries in different facilities. DOD data were used to generate facility-specific clinical practice scores for intrapartum and newborn care [24]. As only daytime deliveries were used, it might have overestimated the performance scores.

### Clinical outcomes and covariates

We used two clinical outcomes–PPH and intrapartum asphyxia. In the setting of a BEmONC facility in Bihar, where there are limitations in equipment and clinician competency, the providers used the accepted definition of PPH as blood loss associated with obstetric labor or childbirth of more than 500ml for a vaginal delivery. However, the operational definition of PPH in this setting was, “a provider observing persistent trickling of more than expected blood, or a blood clot that was the size of a fist, or changing pads every 5–15 minutes.” For PPH management, we examined specific steps of fluid or uterotonics administration.

For intrapartum (or birth) asphyxia, we used the WHO definition of “failure to initiate or sustain breathing at birth”. However, for operational purposes the intervention emphasized to identify neonates who did not breathe within the first 30 seconds, with prompt initiation of PPV to make best use of the first minute after birth. The authors recognize that this a departure from standard recommendations, but we adapted it to instill a sense of urgency. For intrapartum asphyxia management, we examined specific steps of drying, warming, clearing airways and PPV as recommended by ILCOR [25]. Facilities in the sample did not have the equipment or laboratory capacity to assist in the diagnosis of asphyxia.

The terminology pertaining to intrapartum birth asphyxia has evolved to objectively define the condition and correctly identify neonates with the condition. The WHO definition is neither predictive of outcome nor does it imply any causation. The ICD-10 categories of P20 “intrauterine hypoxia” and P21 “birth asphyxia” are classified by onset characteristics [26] but do not provide clear diagnostic criteria or threshold values and APGAR scores, fetal acidosis and fetal distress lack specificity. The terms “post-asphyxial encephalopathy” or “hypoxic ischemic encephalopathy” are also used to describe encephalopathy caused due to intrapartum injury [26, 27]. However, recent guidelines from the American Academy of Pediatrics, American College of Obstetrics and Gynecology, and International Cerebral Palsy Task Force recommend against the use of these terms unless intrapartum-related causation can be established [27]. Instead, the term “neonatal encephalopathy” is recommended. In low and middle income countries, where advanced facilities necessary to ascertain intrapartum causation are rarely available in public health centers, and where a sizeable proportion of the births happen without skilled birth attendants, the chances of neonatal encephalopathy occurring as a result of intrapartum hypoxia are much higher [27]. In keeping with the recommendation, and following other studies from very similar settings [28, 29], we used a clinical symptom-based indicator to determine intrapartum asphyxia because it was the most feasible method of diagnosis that could be implemented in the study setting.

**Fig 1:**
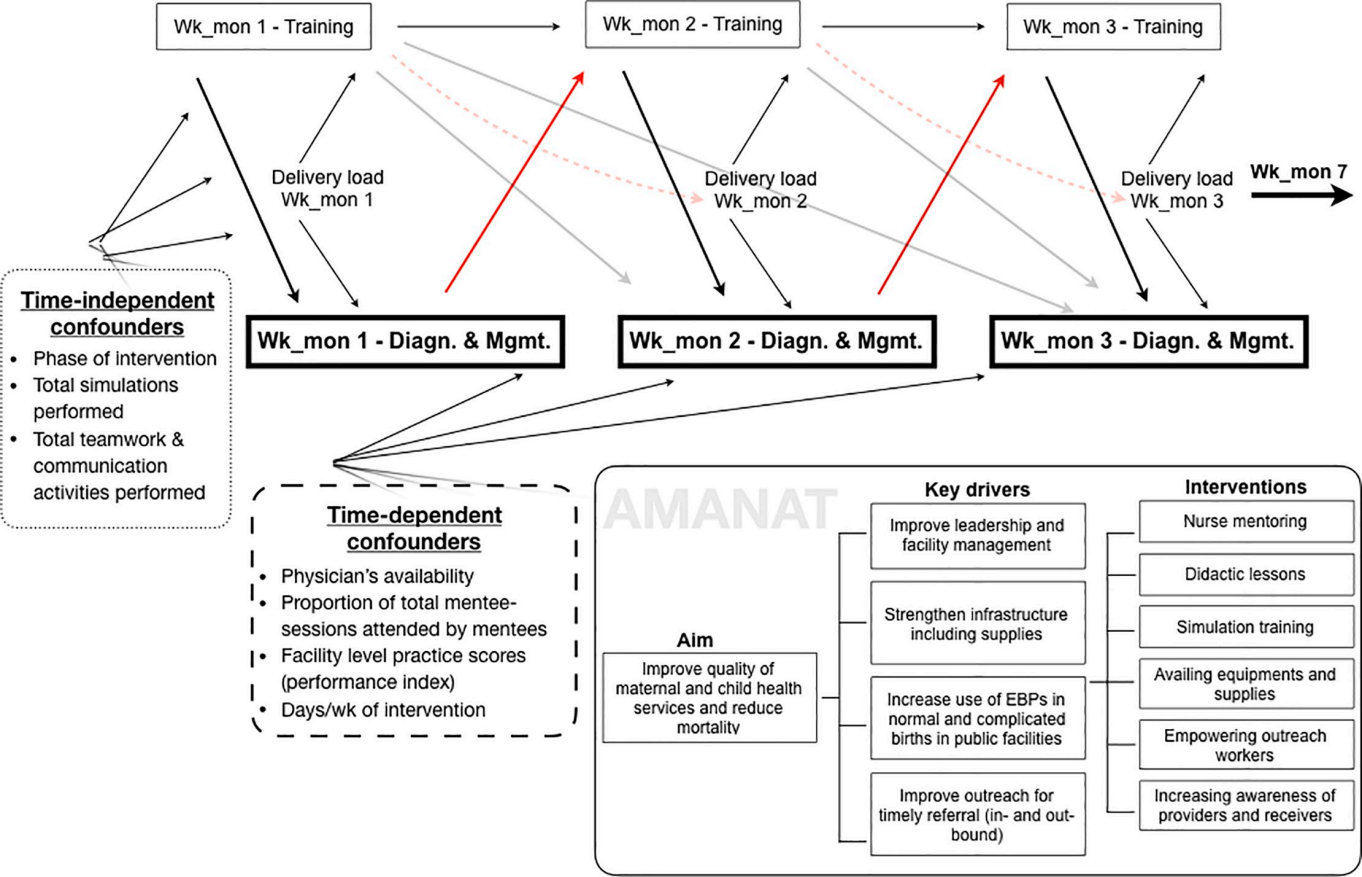
Conceptual framework representing the relationship between week of mentoring and the diagnosis and management of postpartum hemorrhage and intrapartum asphyxia, with potential time-independent and time-dependent confounders. A key driver diagram is also included that gives a broader overview of the overall AMANAT program.

Number of weeks of mentoring per facility was the key variable of interest. There were two sets of covariates: time-dependent and time-independent (Fig 1). Time-independent covariates included phase of intervention, number of complication simulations and T&C activities performed, which accounted for the mentor’s prioritization of activities during mentoring. As this analysis pertained complicated deliveries, only simulation scenarios that involved complications were considered (S1 Appendix). The time-dependent covariates included physician availability during a delivery (in-person or by phone), proportion of total mentee-sessions attended, facility-level practice scores, number of days of mentoring per week, and number of births per week. We calculated availability of a physician as deliveries per mentoring week when a doctor attended a mother or a neonate (or consulted by phone). We measured participation in mentoring activities through the proportion of mentee-sessions that were marked as present (S1 Appendix). The ‘facility level practice scores’ covariate was generated using twenty-three EBPs from DOD data collected before (baseline) and after (endline) intervention (S1 Appendix). As the highest diagnosis was observed around week 5, we assigned the baseline scores to the first 3 weeks and the endline scores from week 4 onwards.

### Statistical analysis

Due to the statewide coverage of the program true controls were not available. To examine the intervention effect, we made a quasi-experimental comparison between-facilities as well as a longitudinal comparison within-facilities over time. For the between-facilities comparison using distinct sets across phases, the proportions in the final week of intervention (intervention effect) were contrasted with the proportions in the first week of the subsequent phase (surrogate controls) that was proximal in time. The respective first and final week comparisons between phases 2 and 1, as well as phases 4 and 3 were concurrent, while for phases 3 and 2 they were five months apart. We estimated the facility specific proportions of diagnosed or managed cases for the first and the final week, which were then averaged across all facilities in that phase.

For the within-facility longitudinal comparison the unit of analysis was facility-week. Using the start and end date of each mentoring week for each facility we aggregated the number of births and complications, and converted the individual-level birth dataset to a repeated observation facility-week longitudinal dataset. As the outcomes were counts with the overall incidences small (<3%) and their variances were greater than the mean, the negative binomial model was preferred. Further, there were many facility-weeks with no complications, when either there were no complications or complications were undiagnosed. In other words, zero counts can be divided into true counts (no complication occurred) and identification errors (complications not diagnosed). These two sets of zeros are statistically identical but generated through two different processes. A facility that fails to identify any complication will always have zero count. However, a facility that identifies complications, will have zero and non-zero counts depending on occurrence. Thus, the number of facilities with zero complications for a facility-week cannot be explained in the same manner as other facility-weeks with one or more identified complications. A standard model would not distinguish between the two processes. Therefore, we utilized the zero-inflated negative binomial model, which includes a binary logistic model to predict the odds that a facility will diagnose or manage complications, while the negative binomial model generated the incidence rate ratios (IRR) for diagnosis or management of complications, per week of mentoring. To account for correlation in the outcomes given that deliveries are clustered in both time and space, we used the sandwich variance estimator, which provides correct standard error for zero inflation models regardless of the correlation [30]. For the diagnosis of complications, we used a one-knot linear spline to model the increasing and decreasing trend (S1 Appendix). Temporal trend in management was linear and was modelled linearly.

Fig 1 shows the complex relationships of exposure-outcome with time-dependent as well as time-independent confounding (S1 Appendix). The thick black arrows from training to diagnosis represent the direct effects of training on diagnosis and management of complications in the concurrent (black) and subsequent (grey) weeks (Fig 1). Mentees performance in week 1 influenced focus of the training in week 2 (e.g. correct diagnosis shifted focus of the training to management), shown by the solid red arrows. These directed paths represent past outcome influencing future exposure. Content of trainings in each week is pre-determined with some flexibility to modify as required, represented by the directed paths from week 1 training to week 2 and so on. Delivery load in the index week influenced time available for training in that week, represented by the directed paths from total births to training weeks. Further, the number of complications identified in a week was also dependent on the number of deliveries in that week, represented by the directed path from total births to diagnosis. Total deliveries in week 1 likely influenced diagnosis and management in week 2 through two pathways: (a) conditional on training time in week 1, which likely affected diagnosis and management in subsequent weeks, and (b) through diagnosis and management in week 1, conditional on training time in week 2. The AMANAT program through infrastructure strengthening likely improved the standing of the facilities in the community and mentoring likely improved service delivery by mentees. The overall improvement in care provided by the facility may increase the delivery load after additional weeks of training, as shown by broken red arrows. The number of complicated simulations run in week 1 likely influenced diagnosis rates in week 1 as well as in subsequent weeks. Furthermore, future diagnosis rates were conditional on learning from past performance of simulations of PPH or intrapartum asphyxia complications. Many of these relationships justify the use of marginal structural models (MSM) to account for time-varying confounding because conventional models will be inadequate [31]. As the results from the MSMs were similar (S1 Table) and the AIC values from MSMs were larger than the individual variable adjusted models (S2 Table), main tables reported the latter.

In a sensitivity analysis, we adjusted the final models with additional confounders, particularly those that are considered risk factors of intrapartum asphyxia, such as premature rupture of membranes, multiple births, preterm birth, low birth weight, obstructed or prolonged labor, cord prolapse, breech presentation, and anemia. The longitudinal trends of these risk factors over the months of mentoring could have varied due to mentoring, thus fulfilling the conditions of confounders.

Two-tailed significance was examined at the 5% level. The final models were restricted to week 7 because the number of facilities receiving >7 weeks of mentoring reduced drastically (Table 1). Comparing weekly proportions from a much smaller subset of facilities with that of the entire pool is misleading. S3 Table presents results without exclusion. We checked the final models for outliers and regression assumptions. We analyzed data using Stata 14.2 (Stata Corp., TX). The Indian Institute of Health Management Research University and the Committee for Human Research at the University of California, San Francisco approved this study.

**Table 1:** Characteristics of deliveries and facilities in the AMANAT nurse-mentoring program in Bihar, India (2015–2017).

Characteristics	n	%^1^
**Total births**	55,938^1^	100
**Deliveries with complications**^2^		
Postpartum hemorrhage	1,291	2.3
Intrapartum asphyxia	1,631	2.9
Pre-eclampsia or eclampsia	302	0.5
Maternal sepsis	83	0.2
**Management of deliveries**		
IV fluids administered	1,595	2.9
Uterotonics administered	1,157	2.1
**Management of newborns**		
Radiant warmer	4,301	7.7
Drying/stimulation	1,866	3.3
Suctioning	1,562	2.8
Positive pressure ventilation	688	1.2
**Birth observation**^3^		
Observed	14,632	26.2
Not observed	32,578	58.2
Partially observed	8,726	15.6
Unknown	2	<0.1
**Phases of AMANAT mentoring program**		
1—May 2015 to Oct 2015	12,341	22.1
2—Sep 2015 to May 2016	18,271	32.7
3—Nov 2015 to Jun 2016	10,088	18.0
4—Jun 2016 to Jan 2017	15,238	27.2
**Physician available during delivery**^4^		
Yes	3,235	5.8
No	52,703	94.2
**# of weeks of training received by facilities (n = 320)**		
1	320^5^	100
2	320	100
3	320	100
4	319	99.7
5	317	99.1
6	312	97.5
7	271	84.7
8	79	24.7
9	3	0.09

^1^ Percentage of total births unless the denominator is mentioned alongside the indicator.

^2^ If a delivery had both PPH and intrapartum asphyxia, it was counted in both of these categories, i.e., the categories are not mutually exclusive.

^3^ Observed: all stages of delivery occurred when a mentor was present in the facility; partially observed: only part of the delivery occurred when a mentor was present in the facility; unobserved: delivery occurred in the absence of a mentor.

^4^ A physician was either physically present or available on call.

^5^ The numbers are cumulative.

## Results

A total of 55,938 deliveries were recorded in 320 facilities during the mentoring period. Of these, 1,291 (2%) had PPH (half of which were atonic) and 1,631 (3%) had intrapartum asphyxia (Table 1). Few had preeclampsia/eclampsia [n = 302 (0.5%)] or sepsis [n = 83 (0.2%)]. More than a quarter of deliveries occurred when mentors were present (observed) in facilities, and 58% occurred outside work hours (not observed). Eighty-five percent of facilities received at least seven weeks of mentoring.

The total number of deliveries occurring in individual facilities over the entire mentoring weeks ranged from 23 to 642, with a median of 159 [interquartile range (IQR): 100, 223] (Table 2). The average number of mentoring days per facility was 39 (SD: 5). On average, facilities performed 19 (SD: 10) maternal, 10 (SD: 5) neonatal simulations and 7 (SD: 6) T&C activities. Average staff attendance in mentoring sessions was 81% (SD: 11%). Facility level intrapartum and newborn practice scores improved from baseline to endline.

**Table 2:** Facility-level characteristics across the duration of the AMANAT nurse-mentoring program in Bihar, India (2015–2017).

		Mean (SD)^1^	Median (IQR)^1^	Min—Max
Number of deliveries per facility in all weeks of mentoring (count)		175 (98)	159 (100, 223)	23–642
Number of mentoring days received (days)		39 (5)	39 (37, 42)	17–53
Number of maternal complication simulations performed (count)		19 (10)	18 (12, 24)	0–60
Number of neonatal complication simulations performed (count)		10 (5)	9 (6, 12)	0–34
Number of teamwork and communication activities performed (count)		7 (6)	5 (3, 11)	0–27
Proportion of mentee-session attendance^2^ (0–100)		81 (11)	82 (75, 90)	42–100
Facility performance index (on a scale of 100)^3^				
Intrapartum	Baseline	21 (12)	21 (8, 29)	0–67
	Endline	56 (19)	58 (42, 67)	8–100
Newborn	Baseline	43 (12)	42 (35, 50)	8–73
	Endline	69 (16)	71 (58, 79)	0–100

^1^ SD–Standard Deviation, IQR–Interquartile Range i.e. 25^th^ percentile and 75^th^ percentile.

^2^ For example, if there were 4 mentees in a facility and there were 10 training sessions during a mentoring week, the total mentee-sessions for the week was 40. Therefore, 80% attendance would mean 4 mentees were present for 8 of the 10 sessions (= 32 mentee-sessions).

^**3**^ A set of 11 and 12 evidence-based practice indicators were used to generate intrapartum or newborn scores, respectively that range from 0 to 100. Zero indicates none of the scores were performed in the facility and 100 means all of those were performed.

In the between-facility comparisons across phases, diagnosis was always higher in the final week of intervention (PPH: 2.5–5.4%, intrapartum asphyxia: 4.2–5.6%) relative to the first week (PPH: 1.2–2.1%, intrapartum asphyxia: 0.7–3.3%), which tended to be significant, except in a few cases (Table 3). In general, proportions of PPH or intrapartum asphyxia cases that were managed using selected EBPs were also higher after intervention but these are based on small numbers and may not be stable estimates (S4 Table).

**Table 3:** Proportions of postpartum hemorrhage and intrapartum asphyxia in the first and final week of intervention. A comparison between facilities across phases.

Comparison groups (time)		First week		Final week		
*Postpartum hemorrhage*		n^1^	Mean (95% CI)	n^1^	Mean (95% CI)	p-value^2^
First week phase 2 (Sep 2015)	Final week phase 1 (Oct 2015)	78	1.17 (0.42, 1.93)	57	2.51 (1.09, 3.93)	0.07
First week phase 3 (Nov 2015)	Final week phase 2 (May 2016)	76	2.09 (1.17, 3.01)	76	3.01 (1.72, 4.30)	0.25
First week phase 4 (Jun 2016)	Final week phase 3 (Jun 2016)	80	1.66 (0.99, 2.33)	63	5.35 (1.68, 9.02)	0.02
Overall		234^3^	1.64 (1.19, 2.09)	196^3^	3.62 (2.29, 4.95)	0.003
*Intrapartum asphyxia*						
First week phase 2 (Sep 2015)	Final week phase 1 (Oct 2015)	78	0.65 (0.25, 1.04)	57	4.17 (2.57, 5.78)	<0.001
First week phase 3 (Nov 2015)	Final week phase 2 (May 2016)	76	2.57 (1.53, 3.61)	76	4.77 (3.06, 6.49)	0.03
First week phase 4 (Jun 2016)	Final week phase 3 (Jun 2016)	80	3.32 (2.29, 4.35)	63	5.57 (2.60, 8.54)	0.12
Overall		234^3^	2.19 (1.67, 2.71)	196^3^	4.85 (3.62, 6.09)	<0.001

^1^ Number of facilities from which the proportion was estimated. Distinct set of facilities were covered in each phase.

^2^ Unpaired t-test was used to compare the overall mean proportions of complications by phase.

^3^ The number is less than 320 as there are three comparisons of approximately 80 pairs of facilities. There was nothing previous to phase 1 where the first week of phase 1 can be compared with.

**Fig 2:**
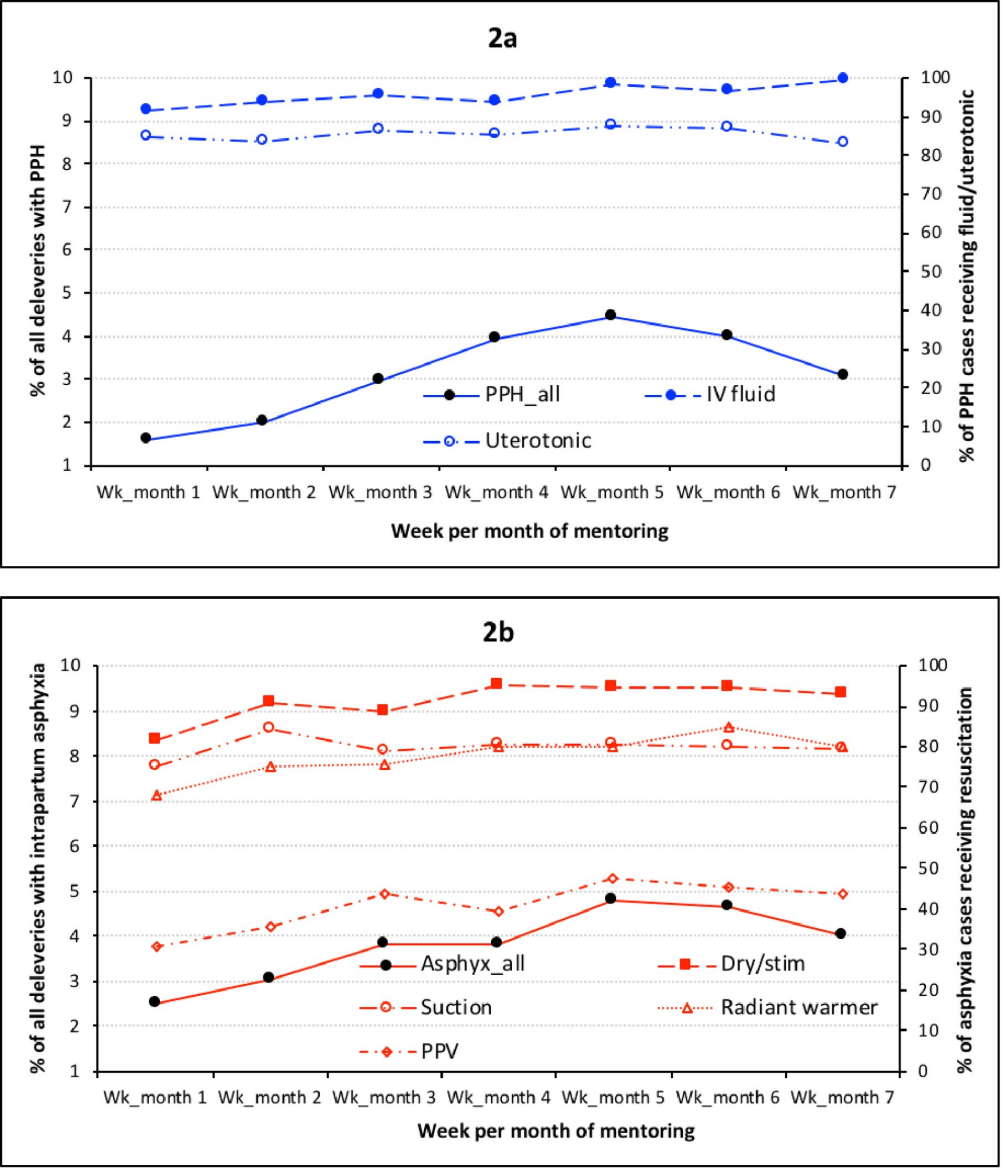
Temporal trend in proportions of diagnosed postpartum hemorrhage (2a) and intrapartum asphyxia (2b) cases and management practices as a proportion of diagnosed cases during the AMANAT mentoring program in Bihar, 2015–2017.

The final longitudinal models had 52,099 deliveries with 1,239 PPH cases, after excluding deliveries with dates inconsistent with arrival and discharge dates and those that occurred outside the days of mentoring (Table 4). The within-facility investigation shows PPH diagnosis among all deliveries increased up to week 5 (from 1.6% to 4.4%), after which they decreased through week 7 (3.1%) and diagnosis was frequent when a mentor was present (Fig 2A). Adjusted IRR demonstrated a 17% increase in PPH incidence [1.17, 95% confidence interval (CI) 1.05, 1.31] associated with each additional week of mentoring up to week 5 and a 14% decline (IRR 0.86, 95% CI: 0.77, 0.97) for weeks 5 through 7 (Table 4). MSM models produced similar IRRs (S1 Table). The odds that a facility will identify a PPH case increased per one-week increase in mentoring, (OR 1.25, 95% CI: 2.17, 3.70).

**Table 4:** Adjusted^1^ incidence rate ratios of changes in the diagnosis and management of postpartum hemorrhage and intrapartum asphyxia in the AMANAT mentoring program in Bihar, India (2015–2017).

	**Postpartum hemorrhage** (1,239/52,099)^2^			
	IRR (95% CI)^3^	p-value	OR (95% CI)^4^	p-value
*Diagnosis*				
Weeks 1–5	1.17 (1.05, 1.31)	0.006	1.25 (2.17, 3.70)	0.006
Weeks 5–7	0.86 (0.77, 0.97)	0.017		
*Management*				
IV fluids^5^	1.01 (0.97, 1.04)	0.688	3.57 (2.70, 4.76)	<0.001
Uterotonic^5^	0.99 (0.95, 1.03)	0.700	2.50 (1.96, 3.13)	<0.001
	**Intrapartum asphyxia** (1,577/52,099)^2^			
*Diagnosis*				
Weeks 1–5	1.21 (1.13, 1.29)	<0.001	6.67 (1.52, 33.33)	0.012
Weeks 5–7	0.91 (0.82, 1.01)	0.073		
*Management*				
Radiant warmer^6^	1.05 (1.01, 1.09)	0.005	1.52 (0.46, 5.00)	0.497
Drying-stimulation^6^	1.05 (1.02, 1.08)	0.003	4.35 (3.23, 5.56)	<0.001
Suctioning^6^	1.03 (0.99, 1.06)	0.127	5.88 (3.70, 9.09)	<0.001
PPV^6^	1.09 (1.02, 1.15)	0.007	-^7^	-

^1^ Adjusted for days per week of nurse-mentoring, total number of births per week, phase of program, physician available, proportion of mentee-sessions attended, facility level practice scores, number of postpartum hemorrhage simulations performed, number of neonatal resuscitation simulations performed, and number of teamwork and communication activities performed. Additionally, the models for management practices were also adjusted for the counts of the respective complications.

^2^ Number of diagnosed cases/Total number of deliveries included in the final model.

^3^ Increase in incidence rate ratios (IRR, 95% confidence interval) for diagnosis of complications, per additional week of mentoring, from the negative binomial part of the zero-inflated negative binomial model.

^4^ Odds ratios (OR) from the logistic part of the zero-inflated negative binomial model, give the odds that a facility will identify complications, per additional week of mentoring.

^5^ Specific management practices relevant for postpartum hemorrhage.

^6^ Specific management practices relevant for intrapartum asphyxia.

^7^ The point estimate for Positive Pressure Ventilation (PPV) is too small [2×10^5^ (95% CI: (1.4×10^4^, 3.3×10^6^)] and the CI is too wide to be of any interpretable importance.

Among all PPH cases, 96% and 84% received IV fluids or uterotonics, respectively. From week 1 through 7, these proportions changed little and in the adjusted models changes per week were not significant (Fig 2A and Table 4).

The diagnosis of intrapartum asphyxia among all livebirths increased from 2.5% in week 1 to 4.8% in week 5, after which it reduced to 4.0% through week 7 (Fig 2B). When a mentor was present, diagnosis generally tended to increase from week to week. Adjusted IRR was 1.21 (95% CI: 1.13, 1.29) for week 1 through 5, followed by non-significant decline (IRR 0.91, 95% CI: 0.82, 1.01), associated with each additional week of mentoring (Table 4). IRRs from the MSM models were similar (S1 Table). In sensitivity analyses, results were practically unchanged to adjustment with other risk factors that are mentioned in the methods. The odds that a facility will diagnose an intrapartum asphyxia case increased with each week of mentoring (OR 6.67, 95% CI: 1.52, 33.33), though the CI was too wide and should be interpreted cautiously.

Seventy-eight percent of the asphyxiated newborns were taken to a radiant warmer, 92% were dried or stimulated, 81% were suctioned and 41% received PPV. From week 1 through 7 of mentoring, asphyxia management improved and adjusted models showed a 5–9 percentage-points increase in radiant warmer use, drying/stimulation and PPV with each additional week of mentoring (Fig 2B, Table 4).

## Discussion

This investigation identified some improvement in the diagnosis of PPH and intrapartum asphyxia in both between- and within-facility comparisons. Comparison between facilities within similar geographies and time generally suggests improvement in diagnosis. Within-facilities over time, diagnosis of PPH and intrapartum asphyxia among all deliveries increased up to week 5, after which it began trending downward. Despite the overall increase in proportions of PPH and intrapartum asphyxia, these were still not on par with levels observed in Southeast Asia and globally, suggesting some complicated deliveries remain undiagnosed [32–34]. For both outcomes, the proportion of diagnosed cases where selected EBPs were used for management either remained stable or increased as diagnosis increased, demonstrating that the absolute number of cases with acceptable management practices kept pace or increased with increased diagnosis. The results also suggest that, among facilities that did not diagnose any PPH or intrapartum asphyxia initially, mentoring enabled providers to begin diagnosing complications. Thus, the nurse-mentoring program appears to have built provider’s capacity to identify PPH and/or intrapartum asphyxia. Once identified, providers seem to be relatively well poised to manage these complications.

Studies from the United States and Canada showed temporal increase in PPH incidence, driven by an increase in uterine atony, changes in demography, maternal comorbidities, or delivery mode [35, 36]. Our results are unlikely to be explained by these factors. We found both increasing and decreasing trends within a relatively short period. It is unlikely that demographic factors reversed directions in this large (>100 million) population [14], in the absence of major events (epidemic, migration etc.). Increase in Caesarean sections cannot explain the results, which are based on vaginal deliveries, nor can delivery load, as the models adjusted for this. Multifetal pregnancy or treatment with magnesium sulfate can overdistend the uterus and compromise contractility, leading to atonic PPH [35]. In this dataset, there were five twin deliveries and three women received magnesium sulfate among those with PPH. Thus, improvements observed in this investigation is likely due to the intervention, although the potential for other explanations remain as we did not have true controls and intervention was not assigned randomly.

Overall proportions of PPH in this study are consistent with another report on Helping Mothers Survive (HMS) Bleeding after Birth [37]. That study assessed blood loss subjectively and reported a decrease in proportion of patients that lost between 500 and 1000 ml of blood but found an increase in the proportion that lost <500 ml, after relative to before training, which could be due to a more accurate assessment after training that shifted patients into different categories [37]. This could be a potential explanation for the downward trend we observed in PPH diagnosis when providers were “over” sensitized to identifying complications in the early weeks, which then normalized to a more accurate assessment over the last couple of weeks. It could also be because routine administration of uterotonics for active management of the third stage of labor (AMTSL) may not have reached the peak by week 5, and may have increased further thereby actually reducing PPH incidence. DOD data on uterotonics use for AMTSL supports this observation (38% at baseline to 71% at endline), though the data to track usage by week were not available. A systematic review reported insufficient evidence to suggest simulation training improves NR [38]. However, other reports from Helping Babies Breathe simulation training reported improved knowledge and skills; clinical performance of stimulation, suction, and bag-mask ventilation; and demonstrated positive impact on fresh stillbirth and mortality on the first day of life [39–42]. Evidence on retention of knowledge and skills after training is mixed [41, 43]. The rigorously conducted Better Birth trial in India and HMS program in Tanzania reported decrease in skills after 9–12 months [44, 45], another very small study of physicians suggests retention of PPH-related skills for up to two years [46]. The management of complications, including uterotonic use and NR, observed in this study was comparable to those observed post-intervention in other settings [37, 47, 48]. Given that mentors collected data, we cannot completely rule out systematic overreporting (bias) of outcomes. However, comparability of our results with that of other studies give confidence against such occurrence. Furthermore, if mentors were systematically overreporting complications, it would be unlikely to see a consistent decline precisely timed at week 5 for both of the outcomes.

Among the strengths are the large statewide coverage powering the investigation and lending limited external validity to facilities in similar low-resource settings and readiness. The longitudinal comparison within-facilities enabled examination of trends over time, which a pre-post design would have missed. The analytical strategy minimizes the possibility of residual confounding and strengthens causal inference because several models and a range of covariate adjustment yielded robust results.

Given the operational limits in Bihar we were unable to use objective measures for the diagnosis of PPH (blood loss) and intrapartum asphyxia (APGAR score, umbilical cord pH, neuroimaging, etc.). We tried using a calibrated obstetric drape to quantify blood loss; however, we could not support universal use, as there were concerns about cleaning, re-use and infections. To establish intrapartum causation of asphyxia more accurately, postnatal neuroimaging or blood gas analysis are needed, which were not available in Bihar, and we acknowledge this as a limitation.

Additionally, we did not have true control facilities and addressed this limitation by using both between- and within-facility comparisons. Another challenge we had was related to measurement of time, which is critical for an asphyxiated infant. Simply noting a specific step to resuscitate an infant was performed with no reference to time portrays an incomplete picture of case management. A related study identified several barriers to clinical urgency among mentees, including poor understanding of the indications (e.g., immediate versus delayed cord clamping, significance of effective ventilation within 60 seconds) [49]. There is also a possibility of reporting bias, as FIS data were collected by nurse mentors, and may not reflect adoption of EBPs by mentees, exclusively, the chances of which are minimal for reasons discussed above. Overreporting will bias the results if it is differential. In other words, overreporting has to be only in certain type of facilities, i.e., those with more or less weeks of mentoring, not both. If overreporting is randomly distributed across all facilities (i.e., non-differential), it will affect significance, but not point estimates [50]. Likewise, non-identification of complications is unlikely to be restricted to facilities with zero counts for all mentoring weeks but scattered across all 320 facilities as it does not depend on facilities but specific provider skills as well as case severity. If non-identification was spread across all facilities and it was non-differential by exposure, significance and not the point estimates will be affected [50]. Finally, we did not have adequate numbers for other important complications such as preeclampsia and sepsis. Global estimates suggest these are also severely underreported.

## Conclusion

During the AMANAT program there was an increase in the diagnosis of PPH, which decreased somewhat during the last two weeks. At baseline, the majority of the PPH cases were managed using selected EBPs, which remained largely unchanged throughout the program. Diagnosis and management of intrapartum asphyxia using selected EBPs improved with duration of mentoring. Diagnosis of PPH and intrapartum asphyxia in public facilities in Bihar is still not on par with regional or international levels. Thus, continued efforts to improve providers’ ability to recognize and act on these important causes of maternal and newborn mortality are needed. In order to sustain the gains achieved through this program, in the next phase of intervention, champion mentees were identified from facilities, then trained to serve as mentors and continue these activities in their respective facilities. This study also provides empirical evidence that, following identification, providers demonstrated the capacity to appropriately manage PPH and intrapartum asphyxia.

## Supporting information

S1 Table Comparison of results from different diagnosis models to demonstrate robustness. (DOCX)

S2 Table A comparison of the model fit statistics of the diagnosis models for PPH and intrapartum asphyxia reported in Table 3. (DOCX)

S3 Table Adjusted^1^ incidence rate ratios of changes in the diagnosis of PPH and intrapartum asphyxia and in management practices per additional week of AMANAT nurse-mentoring in primary health facilities in Bihar, India (2015–2017). The results are for weeks 1 through 9 of mentoring without any exclusion.
(DOCX)

S4 Table Proportions of PPH and intrapartum asphyxia cases that were managed effectively in the first and final week of intervention. A comparison between facilities across phases.
(DOCX)

S1 Appendix Supplementary information. (DOCX)
