# Supplementary material for: Diagnosis and management of postpartum hemorrhage and intrapartum asphyxia in a quality improvement initiative using nurse-mentoring and simulation in Bihar, India
Source: PLoS One. 2019 Jul 5;14(7):e0216654. doi: 10.1371/journal.pone.0216654 (PMC6611567; doi:10.1371/journal.pone.0216654)
Supplement: S1 Table — (DOCX) [file pone.0216654.s001.docx]

## S1 Table

**S1 Table. Comparison of results from different diagnosis models to demonstrate robustness.**

|  | **IRR (95% CI)**^1^ | | | |
| --- | --- | --- | --- | --- |
| **Models** | ***Postpartum hemorrhage*** | | ***Intrapartum asphyxia*** | |
|  | Spline week 1-5 | Spline week 5-7 | Spline week 1-5 | Spline week 5-7 |
| **GEE Negative binomial model**^2^ |  |  |  |  |
| Sandwich variance estimator | 1.21 (1.11, 1.31) | 0.88 (0.79, 0.99) | 1.22 (1.14, 1.31) | 0.89 (0.80, 0.99) |
| Bootstrap variance estimator | 1.21 (1.12, 1.30) | 0.88 (0.76, 1.02) | 1.22 (1.13, 1.32) | 0.89 (0.80, 0.99) |
|  |  |  |  |  |
| **GEE Zero-inflated negative binomial model** |  |  |  |  |
| Cluster sandwich variance estimator | 1.17 (1.05, 1.31) | 0.86 (0.77, 0.97) | 1.21 (1.13, 1.29) | 0.91 (0.82, 1.01) |
| Bootstrap variance estimator | 1.18 (1.07, 1.30) | 0.90 (0.75, 1.07) | 1.24 (1.15, 1.34) | 0.92 (0.80, 1.05) |
|  |  |  |  |  |
| **Marginal structural models**^3^ |  |  |  |  |
| Cluster sandwich variance estimator | 1.16 (1.05, 1.29) | 0.81 (0.72, 0.91) | 1.16 (1.09, 1.24) | 0.86 (0.78, 0.96) |
| Bootstrap variance estimator | 1.14 (1.04, 1.25) | 0.85 (0.72, 1.01) | 1.18 (1.09, 1.28) | 0.88 (0.78, 0.99) |

^1^ Increase in incidence of complication per week of AMANAT training. The models are adjusted for days per week of training, total number of birth per week, phase of intervention, physician available to consult, proportion of mentee-sessions attended, facility level practice scores, number of complication simulations run (maternal for PPH and neonatal for intrapartum asphyxia) and number of teamwork and communication activities performed.

^2^ Unstructured correlation assumed.

^3^ The marginal structural models were adjusted for inverse probability weights only.
