# Supplementary material for: Diagnosis and management of postpartum hemorrhage and intrapartum asphyxia in a quality improvement initiative using nurse-mentoring and simulation in Bihar, India
Source: PLoS One. 2019 Jul 5;14(7):e0216654. doi: 10.1371/journal.pone.0216654 (PMC6611567; doi:10.1371/journal.pone.0216654)
Supplement: S2 Table — (DOCX) [file pone.0216654.s002.docx]

**S2 Table**

**S2 Table. A comparison of the model fit statistics of the diagnosis models for PPH and intrapartum asphyxia reported in table 3.**

|  | ***Postpartum hemorrhage*** | | ***Intrapartum asphyxia*** | |
| --- | --- | --- | --- | --- |
|  | Log likelihood | AIC | Log likelihood | AIC |
| **GEE Zero-inflated negative binomial model** |  |  |  |  |
| ***Cluster sandwich variance estimator*** | ***-1887.1*** | ***3810.1*** | ***-2063.9*** | ***4163.8*** |
| Bootstrap variance estimator | -1910.8 | 3855.5 | -2096.0 | 4226.0 |
|  |  |  |  |  |
| **Marginal structural models** |  |  |  |  |
| Cluster sandwich variance estimator | -1989.4 | 3992.8 | -2288.2 | 4590.3 |
| Bootstrap variance estimator | -2018.7 | 4049.3 | -2355.2 | 4722.3 |
