# Supplementary material for: Diagnosis and management of postpartum hemorrhage and intrapartum asphyxia in a quality improvement initiative using nurse-mentoring and simulation in Bihar, India
Source: PLoS One. 2019 Jul 5;14(7):e0216654. doi: 10.1371/journal.pone.0216654 (PMC6611567; doi:10.1371/journal.pone.0216654)
Supplement: S3 Table — The results are for weeks 1 through 9 of mentoring without any exclusion. (DOCX) [file pone.0216654.s003.docx]

**S3 Table**

**S3 Table. Adjusted^1^ incidence rate ratios of changes in the diagnosis of PPH and intrapartum asphyxia and in management practices per additional week of AMANAT nurse-mentoring in primary health facilities in Bihar, India (2015 – 2017). The results are for weeks 1 through 9 of mentoring without any exclusion.**

|  | ***Postpartum hemorrhage*** | | ***Intrapartum* *asphyxia*** | |
| --- | --- | --- | --- | --- |
|  | IRR (95% CI)^2^ | p-value | IRR (95% CI)^2^ | p-value |
| ***Diagnosis*** |  |  |  |  |
| Slope week 1 – 5 | 1.16 (1.04, 1.30) | 0.01 | 1.20 (1.12, 1.28) | <0.001 |
| Slope week 5 – 9 | 0.90 (0.81, 1.00) | 0.054 | 0.96 (0.87, 1.06) | 0.430 |
| ***Management*** |  |  |  |  |
| IV fluids^3^/ Radiant warmer^4^ | 1.02 (0.98, 1.05) | 0.347 | 1.05 (1.02, 1.09) | 0.002 |
| Uterotonic^3^ / Drying-stimulation^4^ | 1.00 (0.96, 1.04) | 0.972 | 1.05 (1.02, 1.08) | 0.001 |
| Suctioning^4^ | - |  | 1.02 (0.99, 1.06) | 0.152 |
| Positive pressure ventilation^4^ | - |  | 1.10 (1.04, 1.17) | 0.002 |

^1^ Adjusted for days per week of nurse-mentoring, total number of births per week, phase of program, physician available, proportion of mentee-sessions attended, facility level practice scores, number of postpartum hemorrhage simulations performed, number of neonatal resuscitation simulations performed, and number of teamwork and communication activities performed. The models for management practices were also adjusted for the counts of the respective complications.

^2^ Increase in incidence rate ratios (IRR, 95% confidence interval) of diagnosis of complications, per additional week of AMANAT nurse-mentoring, from the negative binomial part of the zero-inflated negative binomial model.

^3^ Management practices relevant for postpartum hemorrhage.

^4^ Management practices relevant for intrapartum asphyxia.
