# Supplementary material for: Diagnosis and management of postpartum hemorrhage and intrapartum asphyxia in a quality improvement initiative using nurse-mentoring and simulation in Bihar, India
Source: PLoS One. 2019 Jul 5;14(7):e0216654. doi: 10.1371/journal.pone.0216654 (PMC6611567; doi:10.1371/journal.pone.0216654)
Supplement: S4 Table — A comparison between facilities across phases. (DOCX) [file pone.0216654.s004.docx]

**S4 Table**

**S4 Table. Proportions of PPH and intrapartum asphyxia cases that were managed effectively in the first and final week of intervention. A comparison between facilities across phases.**

| **Comparison groups (time)** | | **First week** | | **Final week** | |  |
| --- | --- | --- | --- | --- | --- | --- |
| *IV fluids* |  | n^1^ | Mean (95% CI) | n^1^ | Mean (95% CI) | p-value^2^ |
| First week phase 2 (Sep 2015) | Final week phase 1 (Oct 2015) | 19 | 84 (66, 102) | 22 | 100 (100, 100) | 0.05 |
| First week phase 3 (Nov 2015) | Final week phase 2 (May 2016) | 24 | 88 (76, 99) | 28 | 98 (95, 102) | 0.05 |
| First week phase 4 (Jun 2016) | Final week phase 3 (Jun 2016) | 27 | 96 (89, 104) | 16 | 100 (100, 100) | 0.45 |
| Overall | | 70 | 90 (84, 97) | 66 | 99 (98, 101) | 0.009 |
| *Uterotonic* |  |  |  |  |  |  |
| First week phase 2 (Sep 2015) | Final week phase 1 (Oct 2015) | 19 | 89 (74, 105) | 22 | 82 (66, 98) | 0.48 |
| First week phase 3 (Nov 2015) | Final week phase 2 (May 2016) | 24 | 79 (63, 96) | 28 | 82 (67, 97) | 0.05 |
| First week phase 4 (Jun 2016) | Final week phase 3 (Jun 2016) | 27 | 84 (70, 98) | 16 | 87 (74, 100) | 0.77 |
| Overall | | 70 | 84 (75, 92) | 66 | 83 (75, 92) | 0.92 |
| *Radiant warmer* |  |  |  |  |  |  |
| First week phase 2 (Sep 2015) | Final week phase 1 (Oct 2015) | 15 | 60 (32, 88) | 32 | 84 (72, 96) | 0.06 |
| First week phase 3 (Nov 2015) | Final week phase 2 (May 2016) | 32 | 65 (48, 81) | 36 | 82 (69, 95) | 0.09 |
| First week phase 4 (Jun 2016) | Final week phase 3 (Jun 2016) | 43 | 72 (58, 85) | 23 | 70 (51, 90) | 0.89 |
| Overall | | 90 | 67 (58, 77) | 91 | 80 (72, 88) | 0.05 |
| *Drying-stimulation* |  |  |  |  |  |  |
| First week phase 2 (Sep 2015) | Final week phase 1 (Oct 2015) | 15 | 80 (57, 103) | 32 | 90 (80, 99) | 0.35 |
| First week phase 3 (Nov 2015) | Final week phase 2 (May 2016) | 32 | 74 (59, 89) | 36 | 97 (92, 103) | 0.003 |
| First week phase 4 (Jun 2016) | Final week phase 3 (Jun 2016) | 43 | 95 (90, 101) | 23 | 91 (79, 104) | 0.49 |
| Overall | | 90 | 85 (78, 92) | 91 | 93 (88, 98) | 0.07 |
| *Suctioning* |  |  |  |  |  |  |
| First week phase 2 (Sep 2015) | Final week phase 1 (Oct 2015) | 15 | 81 (61, 101) | 32 | 94 (85, 103) | 0.15 |
| First week phase 3 (Nov 2015) | Final week phase 2 (May 2016) | 32 | 80 (66, 95) | 36 | 74 (60, 89) | 0.56 |
| First week phase 4 (Jun 2016) | Final week phase 3 (Jun 2016) | 43 | 68 (54, 82) | 23 | 67 (47, 88) | 0.97 |
| Overall | | 90 | 74 (66, 83) | 91 | 79 (71, 88) | 0.40 |
| *Positive pressure ventilation* |  |  |  |  |  |  |
| First week phase 2 (Sep 2015) | Final week phase 1 (Oct 2015) | 15 | 33 (6, 60) | 32 | 30 (15, 45) | 0.79 |
| First week phase 3 (Nov 2015) | Final week phase 2 (May 2016) | 32 | 27 (11, 42) | 36 | 46 (29, 63) | 0.10 |
| First week phase 4 (Jun 2016) | Final week phase 3 (Jun 2016) | 43 | 41 (27, 56) | 23 | 49 (29, 70) | 0.52 |
| Overall | | 90 | 35 (25, 45) | 91 | 41 (31, 51) | 0.37 |

^1^ Number of facilities from which the proportion was estimated. Distinct set of facilities were covered in each phase.

^2^ Unpaired t-test was used to compare the overall mean proportions of complications by phase.
