# Supplementary material for: Diagnosis and management of postpartum hemorrhage and intrapartum asphyxia in a quality improvement initiative using nurse-mentoring and simulation in Bihar, India
Source: PLoS One. 2019 Jul 5;14(7):e0216654. doi: 10.1371/journal.pone.0216654 (PMC6611567; doi:10.1371/journal.pone.0216654)
Supplement: S1 Appendix — (DOCX) [file pone.0216654.s005.docx]

**S1 Appendix – Supplementary information**

# Diagnosis and management of obstetric and neonatal complications in a quality improvement initiative using nurse-mentoring and simulation in Bihar, India

## Covariates

The program was implemented in four phases and to account for differences across phases we adjusted for phases of intervention. For example, nurse mentor pairs differed by phases of intervention. Phase 1 mentors were the same as the Phase 3, and high performing mentors from Phases 2 and 3 were selected for Phase 4. As this analysis focuses specifically on diagnosis of complications, only the simulations with relevant complications were included i.e. the PPH models were adjusted with the counts of maternal complication simulations and intrapartum asphyxia models were adjusted with the counts of neonatal complication simulations. Normal spontaneous vaginal delivery simulations lacking obstetric or neonatal complications were excluded.

Because mentees rotated between mentoring sessions and clinical duties in a day, an overall present or absent status of a mentee for a day would be misleading. For this reason, we created total mentee-sessions and estimated the proportion of the total sessions marked as present. For example, if facility A had 10 mentees and week 1 had 40 total sessions, there will be a maximum of 400 mentee-sessions in that facility-week. Of these, if 8 mentees were present for all 40 sessions, one was present for 30 sessions, and one was present for 10 sessions, this would equate to 90% attendance [(320+30+10)/400]. This percentage was our variable for adjustment in the final models.

Intrapartum and newborn care composite scores were calculated using a set of evidence based practice indicators collected from direct observations of deliveries. The intrapartum score was comprised of 11 EBPs: (1) checked blood pressure before delivery, (2) checked pulse before delivery, (3) monitored fetal heart sound in first stage of labor, (4) monitored fetal heart sound in second stage of labor, (5) checked for cord around fetus’s neck, (6) uterotonics administered in the third stage of labor, (7) controlled cord traction performed, (8) uterus massaged, (9) checked for completeness of placenta and (10) checked for completeness of membranes and (11) delivery attendant wore gloves. The newborn score was comprised of 12 EBPs: (1) infant placed on mother’s abdomen immediately after delivery, (2) cord pulsation checked before clamping, (3) cord clamped after waiting for one-two minutes, (4) sterile cord clamps used, (5) cord was cut using sterile blade, (6) skin-to-skin contact initiated, (7) eyes wiped with sterile gauze, (8) newborn dried, (9) newborn wrapped in clean dry cloth, (10) cord stump was left clean, (11) weight taken at birth and (12) infant was breast fed within an hour after birth. Individual EBP indicators were assigned a score of 1 if performed as recommended, otherwise, it was scored 0. Given the significance of uterotonic to prevent postpartum hemorrhage the expert panel recommended “uterotonics administered in the third stage of labor” be given twice the weight compared to other individual indicators. The indicator specific scores were aggregated to obtain a total score for each delivery, which was rescaled by dividing it with the maximum possible score. After rescaling, the final scores ranged between 0 and 100, where a 0 refers to none and 100 refers to all EBPs being performed appropriately for a delivery. The scores for individual deliveries in a facility were averaged to obtain a facility level overall score.

## Statistical analysis

Locally-weighted scatterplot smoothing showed that the trend in the rate of diagnosis of complications over weeks of mentoring was non-linear. We modelled the non-linear trends using splines. We used a one-knot linear spline to model the initial increasing trend followed by a downward trend in the last couple of weeks. Figure 2 shows that the increasing trend in diagnosis reached an inflection point around week 5 for both outcomes, hence the knot was placed at that point. The trend in management was largely linear and modelled linearly (Figure 2).

A directed acyclic graph (Figure 1) was drawn to conceptualize the exposure-outcome relationship in the context of time-dependent and time-independent confounding. Figure 1 displays the relationships for weeks 1 through 3 but the same pattern is repeated for the entire duration of the program. The figure shows a few complex relationships, such as diagnosis in week one (past outcome) influencing training (future exposure) in week two or training in week one affecting diagnosis in week three (i.e., after a lag). Since the AMANAT training was done in-situ, facility delivery load determined the time available for training. As a consequence, conventional regression models may not fully account for time-varying confounding. Therefore, we used marginal structural models (MSM) to fully account for time-varying confounding and estimated the inverse probability weights (i.e., inverse of the probability that a facility received mentoring conditional on time-dependent and time-independent factors) and adjusted the final models accordingly. Because there was little variation in the number of weeks of trainings across facilities, we used days/week of training to estimate the inverse probability weights.

**RESULTS**

## Postpartum hemorrhage

From week 1 through 7, proportion of PPH cases that received IV fluids increased, reaching almost universal levels, while the proportion receiving uterotonics remained largely stable; neither of these demonstrated a change per week in the adjusted models (Figure 2a and Table 3). However, the odds of being a facility not performing any of the two PPH management steps reduced with mentoring (OR 0.28, 95% CI: 0.21, 0.37 for IV fluids, OR 0.40, 95% CI: 0.32, 0.51 for uterotonics, Table 3).

## Intrapartum asphyxia

From week 1 through 7 of mentoring, asphyxia management practices increased by >10 percentage points by week (Figure 2b). Adjusted models showed a 5-9 percentage-point increase in radiant warmer use, drying/stimulation and PPV with each week of mentoring (Table 3). The odds that a facility failed to dry/stimulate or suction asphyxiated newborns decreased with additional week of mentoring; this association was not observed for radiant warmer usage (Table 3).

The dispersion parameter for the PPH diagnosis model was 0.79 (95% CI: 0.55, 1.14), signifying that the outcome is over-dispersed and, as the interval does not contain 0, the negative binomial model fits better than Poisson. The dispersion parameters for the IV fluid model was 0.14 (95% CI: 0.09, 0.21) and for the uterotonic model was 0.18 (95% CI: 0.12, 0.27). The dispersion parameter for the intrapartum asphyxia diagnosis model was 0.41 (95% CI: 0.30, 0.55). The dispersion parameters for the four NR management steps were: radiant warmer – 0.30 (95% CI: 0.18, 0.49), drying/stimulation – 0.24 (95% CI: 0.14, 0.39), suction – 0.28 (95% CI: 0.17, 0.46), and PPV – 0.64 (95% CI: 0.38, 1.07).
